# Editorial: The mechanism and clinical application of neuroendocrine hormones in infertility-related diseases

**DOI:** 10.3389/fendo.2023.1206584

**Published:** 2023-06-09

**Authors:** Yajing Liu

**Affiliations:** First Affiliated Hospital of Anhui Medical University, Hefei, China

**Keywords:** neuroendocrine hormones, reproductive disease, PCOS (polycystic ovarian syndrome), Gonadotropin-releasing hormone, epidermal growth factor receptor

The neuroendocrine hormones were found in follicular fluid and ovarian granulosa cells, which indicated their role in infertility-related diseases. It is important to investigate and understand these neuroendocrine hormones on infertility related diseases. The current Research Topic provide new advancements by highlighting recent research findings of neuroendocrine hormones in reproductive endocrine diseases.

Wu et al. investigated the effect of weight loss on the outcomes of assisted reproductive therapy, and related hormones in granulosa cells in clinical PCOS patients. They found that weight loss (>5kg) might affect the secretion of neuroendocrine hormones, and insulin resistance of ovarian granulosa cells, and further to improve the outcomes of clinically assisted reproductive therapy in obese PCOS patients.

The study by Wang et al. showed that function of the hypothalamic–pituitary–testicular (HPT) axis was impaired, and there was a significant reduction in the related hormone level in Wilson’s disease (WD) mice. Furthermore, copper deposition might induce apoptosis and inhibit ERK signaling pathway, and further cause the decrease of related neuroendocrine hormones level in WD mice model. Their study suggested that neuroendocrine hormones played an important role in the WD, and could also provide data for the damage to the male reproductive function caused by copper pollution.

Zhang et al. evaluated the association between epidermal growth factor receptor (EGFR) and polycystic ovarian syndrome (PCOS) by clinical sample and animal model. They found that reduction of EGFR expression in PCOS mice model ameliorated ovulation of the ovaries, and EGFR inhibitor treatment improved the corpopa lutea number of PCOS. Their results indicated that EGFR played a vital role in the occurrence and progression of PCOS by regulating granulosa cell proliferation and oocyte development, suggesting that EGFR inhibition has broad application prospect in clinical treatment of PCOS.

The study by Yu et al. examined the efficacy of hormone replacement therapy (HRT) with gonadotropin-releasing hormone (GnRH) agonist preconditioning in infertility women for male reasons. They found that pretreatment with GnRH agonist improved the outcomes of clinically-assisted reproductive therapy in infertility women for male reasons.

## Author contributions

The author confirms being the sole contributor of this work and has approved it for publication.

